# Evaluation of Ertapenem use with Impact Assessment on Extended-Spectrum Beta-Lactamases (ESBL) Production and Gram-Negative resistance in Singapore General Hospital (SGH)

**DOI:** 10.1186/1471-2334-13-523

**Published:** 2013-11-06

**Authors:** Cheryl Li-Ling Lim, Winnie Lee, Amanda Ling-Chiu Lee, Lisa Ting-Ting Liew, Szu Chin Nah, Choon Nam Wan, Maciej Piotr Chlebicki, Andrea Lay-Hoon Kwa

**Affiliations:** 1 Department of Pharmacy, Singapore General Hospital, Block 8 Level 2, Outram Road, Singapore 169608, Singapore; 2 Department of Infectious Diseases, Singapore General Hospital, Academia Level 3, 20 College Road, Singapore 169865, Singapore

**Keywords:** Ertapenem evaluation, ESBL production, Gram-negative resistance

## Abstract

**Background:**

Ertapenem (preferred choice for ESBL-producing organisms) use exhibited an increasing trend from 2006 to 2008. As extensive use of ertapenem might induce the mutation of resistant bacteria strains to ertapenem, we aimed to assess the appropriateness and impact of ertapenem-use, on ESBL production, the trends of gram-negative bacterial resistance and on the utilization of other antibiotics in our institution.

**Methods:**

Inpatients who received a dose of ertapenem during 1 January 2006 to 31 December 2008, were reviewed. Pertinent patient clinical data was extracted from the pharmacy databases and assessed for appropriateness based on dose and indication. Relevant data from Network for Antimicrobial Resistance Surveillance (Singapore) (NARSS) was extracted, to cross-correlate with ertapenem via time series to assess its impact on hospital epidemiology, trends of gram-negative resistance and consumption of other antibiotics from 2006 to mid-2010.

**Results:**

906 cases were reviewed. Ertapenem therapy was appropriate in 72.4% (93.7% success rate). CNS adverse events were noted in 3.2%. Readmission rate (30-day) due to re-infection (same pathogen) was 5.5%. Fifty cases had cultures growing *Pseudomonas aeruginosa* within 30 days of ertapenem initiation, with 25 cases growing carbapenem-resistant *Pseudomonas aeruginosa*.

Ertapenem use increased from 0.45 DDD/100 patient days in 2006 to 1.2 DDD/100 patient days in mid-2010. Overall, the increasing trend of ertapenem consumption correlated with 1) increasing incidence-densities of ciprofloxacin-resistant/cephalosporin-resistant *E. coli* at zero time lag; 2) increasing incidence-densities of ertapenem-resistant *Escherichia. coli* and *Klebsiella spp.* at zero time lag; 3) increasing incidence-density of carbapenem-resistant *Pseudomonas aeruginosa*, at zero time lag.

Increasing ertapenem consumption was significantly correlated with decreasing consumption of cefepime (R^2^ = 0.37344) 3 months later. It was significantly correlated with a decrease in imipenem consumption (R^2^ = 0.31081), with no time lag but was correlated with subsequent increasing consumption of meropenem (R^2^ = 0.4092) 6 months later.

**Conclusion:**

Ertapenem use was appropriate. Increasing Ertapenem consumption did not result in a decreasing trend of ESBL producing enterobacteriaceae and could result in the selection for multi-drug resistant bacteria.

## Background

Antibiotic options in the treatment of extended spectrum beta-lactamases (ESBL)-producing organisms are extremely limited. Carbapenems are the treatment of choice for serious infections due to ESBL producing organisms [1]. Ertapenem, which belongs to the class of carbapenems, possesses antibacterial activity against ESBL-producing enterobacteriaceae and *Haemophilus* species and not the nonfermenters [2]. Hence, it is the preferred carbapenem choice for infections caused by ESBL-producing organisms currently.

Ertapenem has a longer half-life than the older carbapenems, hence allowing for once-daily administration [3,4]. This allows the patients to utilize the convenience of Outpatient Parenteral Antibiotic Therapy (OPAT) clinic in our institution, Singapore General Hospital (SGH), which reduces length of hospitalization in our hospital. Though ertapenem is in the hospital formulary since 2006, written or verbal approval for use has to be sought with the Infectious Diseases physicians. The use of ertapenem exhibited an increasing trend from 2006 to 2008. In July 2009, this prescribing restriction was removed. Henceforth, ertapenem is approved for use by any physicians if it is culture-directed (presence of ESBL-producing enterobacteriaceae evident by 3rd generation cephalosporins resistance) or for empiric treatment of suspected ESBL infections in patient who has a history of ESBL infections in the past 90 days. Every ertapenem case will be subjected to within 24-hour concurrent feedback audit by Antimicrobial Stewardship team.

In SGH, where extended spectrum beta-lactamases are prevalent, there is a gradual rise in the use of carbapenems, which could bring about acquired resistance. Resistance to carbapenems is due to changes in outer membrane proteins, efflux pumps and potent metalloenzymes that cause hydrolysis of carbapenems [5]. Thus, with the introduction of ertapenem into the formulary, it was thought that we could preserve the use of the other anti-pseudomonal carbapenems for infections involving *Pseudomonas aeruginosa.*

On the other hand, there are concerns that extensive use of ertapenem may induce the mutation of resistant bacteria strains to ertapenem and select out *Pseudomonas aeruginosa*[5,6] strains that might have cross-resistance to other anti-pseudomonal carbapenems. While some studies have shown that ertapenem use was not associated with carbapenem-resistant *Pseudomonas aeruginosa,* these only looked at pharmacy purchasing databases, which may not necessarily reflect actual ertapenem prescription [7-10]. Moreover, none of the studies evaluated ertapenem use and development of gram-negative bacterial resistance at patient-level.

Hence, we evaluated the appropriateness and outcomes of ertapenem use in SGH from 2006 to 2008 and assessed the impact of ertapenem on the hospital’s epidemiology, especially ESBL production, trends of gram-negative bacterial resistance, and utilization of other antibiotics, from 2006 to mid-2010 (1.5 years after the evaluation of use).

## Methods

### Study design

This study was a single-centered, retrospective study which was approved by the SingHealth Institutional Review Board.

### Inclusion criteria and data collection

Patients were included in the study if they received at least one dose of ertapenem in Singapore General Hospital between 1 January 2006 and 31 December 2008 and those who were solely prescribed ertapenem as OPAT were excluded. Patient demographics and treatment related data (details of ertapenem and prior antibiotic therapy, clinical and microbiological outcomes) were extracted from the hospital’s medical records and pharmacy database.

### Appropriateness of ertapenem use

Ertapenem use was deemed appropriate only if criteria for both indication and dose were met. The appropriate indications for ertapenem include i) definitive therapy for ESBL producing enterobacteriaceae or non-ESBL producing enterobacteriaceae in penicillin allergic patients, where susceptibility to ertapenem is confirmed and there were limited therapeutic options left, and ii) empiric ertapenem therapy for septic patients who had prior hospitalization with positive cultures for ESBL producing enterobacteriaceae within the last 90 days. Enterobacteriaceae, that are ESBL producing, are characterized by their resistance to 3^rd^ generation cephalosporins (e.g. ceftriaxone and ceftazidime) [11]. ESBL production was also confirmed using double-disk diffusion method [1]. Use of ertapenem for i) *Pseudomonas aeruginosa* and *Acinetobacter spp.* infections, ii) primary bacteremia and iii) central nervous system infections (CNS) are defined as being inappropriate. Ertapenem dosing was considered appropriate if dose was adjusted according to patients’ renal function (calculated using the Cockcroft-Gault equation) [12], where dose has to be reduced from 1g once daily to 500mg once daily in patients with creatinine clearance less than 30ml/min.

### Outcome analysis and definitions

The clinical efficacy and safety of ertapenem use was assessed and recorded, from the first day to the last day of ertapenem therapy. Treatment outcomes of ertapenem such as i) presence of clinical improvement and ii) presence of microbiological eradication of causative organism(s) were evaluated. Clinical improvement was defined as normalization of blood counts, inflammatory markers, temperature and vital signs. Microbiological eradication was concluded when there was no further bacterial growth of causative organism(s). Where repeat cultures and sensitivities were not performed, eradication was presumed if the patient was discharged clinically well. Hence, treatment success was defined as i) presence of microbiological eradication (or presumed microbiological eradication) primarily, with and without clinical improvement or ii) presence of clinical improvement (followed by home discharge), if follow up cultures were absent. New infections were defined as infections with different pathogens, that occurred after ertapenem initiation; excluding those that were present prior to ertapenem therapy.

Other outcomes used to assess treatment efficacy include 30-day mortality (from first date of ertapenem use) and discharge status (death, home or nursing home). Hospital readmission and re-infection with the same causative organism within 30 days were also evaluated.

### Impact of ertapenem on micro-ecology and antibiotic utilization

To assess the association of ertapenem consumption with consumption of other broad-spectrum antibiotics, ertapenem utilization over a course of 4.5 years from 2006 to mid-2010 was correlated with the consumption of selected antibiotics (ciprofloxacin, ceftriaxone, cefepime and anti-pseudomonal carbapenems). All utilization data, defined as defined daily dose (DDD) per 100 inpatient-days of antibiotic, which corresponds to the assumed average daily maintenance dose, was obtained from Network for Antimicrobial Resistance Surveillance (Singapore) (NARSS) [13-16]. NARSS was established in December 2005 to conduct prospective surveillance of antibiotic resistance and prescription in Singapore public hospitals, with the aim of analyzing trends over time. Antibiotic resistance and prescription data in NARSS was obtained from the hospital laboratory information systems and pharmacy prescription databases.

Ertapenem consumption over the same period of time was also compared to the incidence density of certain microorganisms per 1000 inpatient-days (PD) reported in NARSS [15-21] to evaluate impact of ertapenem consumption on gram-negative resistance trends. These microorganisms include a) ciprofloxacin-resistant *Escherichia coli* and *Klebsiella spp.,* b) cephalosporin-resistant *Escherichia coli* and *Klebsiella spp.* isolates. Incidence densities of the following microorganisms were measured as number of cases per 10,000 PD: a) ertapenem-resistant *Klebsiella spp.* and *Escherichia coli*, b) carbapenem-resistant *Acinetobacter baumannii,* c) carbapenem-resistant *Pseudomonas aeruginosa*. These microorganisms were reported in NARSS because of their potential adverse impact on nosocomial infections and public health [13-16].

### Statistical analysis

Univariate analysis was done to describe the patients’ demographics. Time series analysis was first performed for trend of antibiotic prescription and antimicrobial resistant bacteria over time; and if significant, cross-correlation analysis was then performed to measure the impact of ertapenem on micro-ecology and antibiotic utilization via STATA version 10 (StataCorp LP, Texas USA).

## Results

### Patient demographics

**Figure 1:**
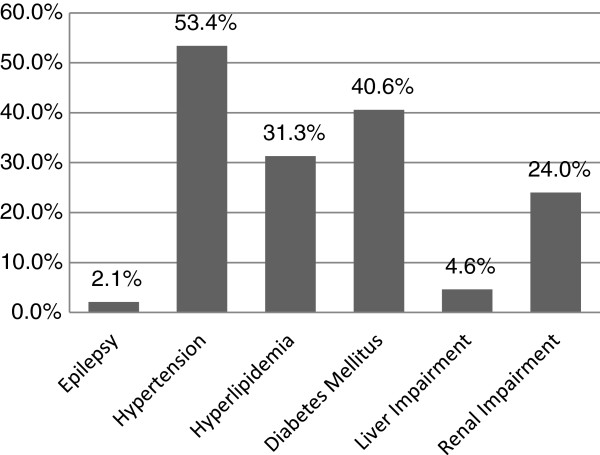
Medical Co-morbidities.

A total of 929 patients were treated with ertapenem between 1 January 2006 and 31 December 2008. Twenty-three patients were excluded because of unavailability of case notes. Hence, only 906 patients were analyzed. The mean duration of hospital stay was 36.55 ± 45.53 days. The median age of cases was 63.00 ± 15.74 years. More than half of the patients had underlying hypertension, diabetes mellitus and/or hyperlipidemia (Figure 1).

**Figure 2:**
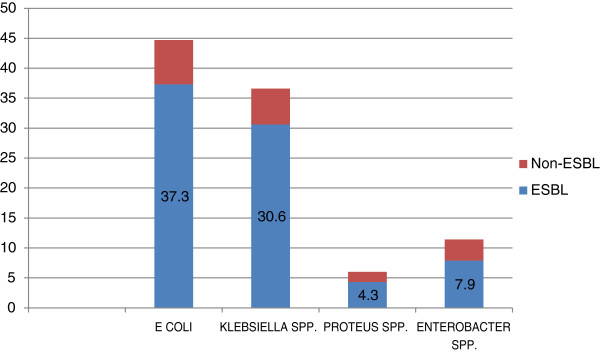
Types of Microorganisms Isolated.

Table 1 described the types of infections for which ertapenem was used in the study patients, and these infections may or may not be appropriate for ertapenem use. Urinary tract infection and bacteremia were the two main infections for ertapenem use (Table 1). Out of the 306 cases that were due to bacteremia, 134 cases were due to primary bacteremia while 172 cases were due to secondary bacteremia with known sources. The main culture sites were: urine (35.2%), blood (33.8%), wound (23.8%), other body fluids (11.1%) and sputum (6.2%). Approximately 7 out of 10 cases had ESBL infections from *Escherichia coli* and *Klebsiella spp.* (Figure 2).

**Table 1:** Types of infections for which ertapenem was used

	**No. of Cases (%)**
Bacteremia	306 (33.8)
Urinary tract infection	337 (37.2)
Intra-abdominal infection	141 (15.6)
Pneumonia	74 (8.2)
Skin and soft tissue infection	145 (16.0)
Others*	64 (7.1)

*Other infections for which ertapenem was used include: line sepsis, osteomyelitis, graft infections, surgical prophylaxis and CNS infections. Patients may have had more than one type of infection for ertapenem use. These infections may or may not be appropriate.for ertapenem use.

### Appropriateness

**Figure 3:**
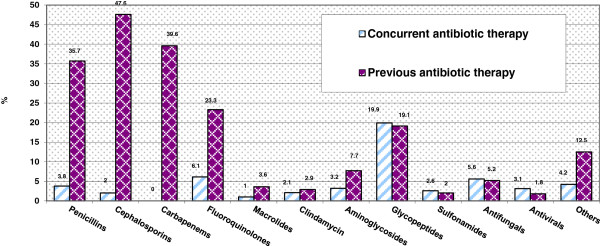
Previous & Concurrent Antibiotic Therapy.

Approximately 72.4% of the cases fulfilled the criteria for appropriate ertapenem use. While 86.0% cases (n = 779) were culture-directed (Table 2), 13.2% and 0.7% of cases were empirical and prophylactic, respectively. Of the culture-directed cases, 46.0% were de-escalation from broader carbapenems to ertapenem (359 out of 779 culture-directed cases). Cephalosporins (47.6%) were the most common beta-lactam antibiotics used before ertapenem, followed by anti-pseudomonal carbapenems (39.6%) such as imipenem and meropenem (Figure 3). As ertapenem is not indicated for surgical prophylaxis, none of the prophylaxis cases were included in the efficacy (under outcomes) analysis. The median duration of ertapenem therapy was 8 (1 – 75) days.

**Table 2:** Details of ertapenem therapy

	**No. of Cases (%)**
**Indication for ertapenem use**	
Empiric	120 (13.2)
Prophylaxis	7 (0.7)
Culture-directed	779 (86.0)
**Dose distribution**	
500 milligrams once daily	149 (16.5)
1 gram every other day	16 (1.8)
1 gram once daily	730 (80.6)
1 gram stat	6 (0.6)
Others^	5 (0.5)
**Median duration of therapy (days) (range)**	8 (1 – 75)
**Prescribed as outpatient antibiotic therapy (OPAT#)**	185 (20.4)
**Other antibiotic use** in the same admission	
Concurrent antibiotic therapy	566 (62.5)
Previous antibiotic therapy	778 (85.9)
Previous beta-lactam use *	754 (83.3)

^Others includes 250 milligrams once daily, 1 gram three times a week.#These patients only include those that continue OPAT from hospitalization.*Examples of beta-lactams include amoxicillin-clavulanic acid, ceftriaxone, cefepime and piperacillin-tazobactam.

### Outcomes analysis

#### Safety of ertapenem treatment

Adverse events related to or suspected to be induced by ertapenem were noted in 46 cases (5.1%), with the majority of those involving the CNS (n = 29) (Table 3). Seizures and confusion occurred in 9 (1.0%) and 20 cases (2.2%) respectively. Of the 9 cases with seizures, 4 cases (44.4%) occurred in patients with underlying epilepsy (Table 3), where only one was inappropriately dosed. In 6 of these 9 cases (66.7%), patients had renal impairment.

**Table 3:** Adverse drug reactions

**Adverse drug reaction**	**No. of Patients (%)**
Seizures	9 (1.0)
Seizures occurring in those with previous history of epilepsy	4 (0.4)
Central nervous system side effects (excluding seizures)	20 (2.2)
Gastrointestinal side effects	3 (0.3)
Others*	10 (1.1)

*Other adverse drug reactions included drug fever, eosinophilia and urticaria.

#### Efficacy

The number of cases which were included for efficacy analysis was 899. Treatment success was observed in 842 cases (93.7%). Out of the 57 treatment failures, 14 cases were prescribed ertapenem for bacteremia; of which 8 and 6 cases were primary and secondary bacteremia respectively. One hundred and two cases (11.3%) had new infections, with 58 cases (56.9%) occurring during ertapenem therapy and 44 cases (43.1%) occurring after discontinuation of therapy. Out of the 102 cases with new infections, 8 and 4 were primary and secondary bacteremia respectively. Thirty-five cases with new infections were polymicrobial in nature.

#### Mortality and re-admission

The proportion of live-discharges was 87.9% (n = 796) (Table 4). Of which, 20.4% (n = 185) were readmitted within 30 days post-discharge with 5.5% (n = 44) being re-infected with the same causative microorganism. Seventy-three (36 primary and 37 secondary) out of 306 cases with bacteremia were readmitted within 30 days. Out of these 73 cases, 3 were readmitted with primary bacteremia and 4 were readmitted with secondary bacteremia. Overall mortality rate within 30 days from the first day of ertapenem treatment was 10.7% (n = 97).

**Table 4:** Mortality and re-admission

**Discharge status**	**Frequency (%)**
Death	97 (10.7)
Home	724 (79.9)
Nursing home	72 (7.9)
Hospital readmission (within 30-day of discharge)	185 (20.4)
Re-infection with the same causative microorganism (in relation to hospital re-admission)	44 (5.5)

#### Resistance development

Fifty cases (5.5%) had cultures growing *Pseudomonas aeruginosa* within 30 days from initiation of ertapenem therapy (Table 5) with 25 cases growing carbapenem-resistant *Pseudomonas aeruginosa*.

**Table 5:** Microorganisms isolated within 30 days from the start date of ertapenem therapy

Microorganism*	**Infection with new microorganism within 30 days of first date of ertapenem use (%)**	**Carbapenem resistance (%)**
*Pseudomonas aeruginosa*	50 (5.5)	25 (2.8)
*Stenotrophomonas maltophila*	21 (2.3)	21 (2.3)**
*Acinetobacter baumannii*	27 (3.0)	20 (2.2)
Enterobacteriaceae (such as *Klebsiella* spp*., E. coli, Proteus* spp.) with ESBL production	41 (4.5)	8 (0.9)

*More than one microorganism was isolated in some cases within 30 days from initiation of ertapenem therapy.

**Carbapenem sensitivity testing was not performed. However, *Stenotrophomonas maltophila* is intrinsically resistant to carbapenems.

### Correlation of ertapenem consumption with incidence of antibiotic-resistant bacteria

Ertapenem use increased from 0.45 DDD/100 PD in 2006 to 1.2 DDD/100 PD in mid-2010. Cross-correlation analysis of ertapenem consumption to microorganisms with significant trend changes in incidence rates from 2006 to mid-2010 were performed and reported in Table 6.

**Table 6:** Correlation of ertapenem consumption with incidence of antibiotic-resistant bacteria

	Correlation Coefficient*	**R**^2*^	P-values*	**Time Lag**
Ciprofloxacin-resistant *E. coli*	0.5349	0.2862	0.111	Zero
Cephalosporin-resistant *E. coli*	0.6665	0.4442	0.035	Zero
Ciprofloxacin-resistant *Klebsiella spp.*	−0.4393	0.0393	0.583	−1 year
Cephalosporin-resistant *Klebsiella spp.*	−0.6752	0.3514	0.071	−6 months
Ertapenem-resistant *E. coli*	0.7144	0.5103	0.020	Zero
Ertapenem-resistant *Klebsiella spp.*	0.5249	0.2755	0.119	Zero
Carbapenem-resistant *Acinetobacter baumannii*	−0.6485	0.0911	0.397	−1 year
Carbapenem-resistant *Pseudomonoas aeruginosa*	0.5648	0.3190	0.089	Zero

*Correlation coefficient, R^2^ and P-values are obtained from cross-correlation analysis.

Incidence-density of ciprofloxacin-resistant *Escherichia coli* increased from 3.2 cases per 1000 PD in 2006 to 3.7 cases per 1000 PD in mid-2010; the same increasing trend was also observed in cephalosporin-resistant *Escherichia coli* (1.6 to 2.0 cases per 1000 PD). Increasing ertapenem consumption correlated, at zero time lag, with increasing incidence-density of 1) ciprofloxacin-resistant *Escherichia coli* (R^2^ = 0.2862) insignificantly and 2) cephalosporin-resistant *Escherichia coli* (R^2^ = 0.4442) significantly. Conversely, ciprofloxacin and ceftriaxone-resistant *Klebsiella spp* incidence densities decreased independently, before the increase in ertapenem consumption. There was approximately 50% decrease in incidence-density of ciprofloxacin-resistant *Klebsiella spp.* (2.2 to 1.1 cases per 1000 PD) and ceftriaxone-resistant *Klebsiella spp.* (2.4 to 1.5 cases per 1000 PD) from 2006 to mid-2010.

**Figure 4:**
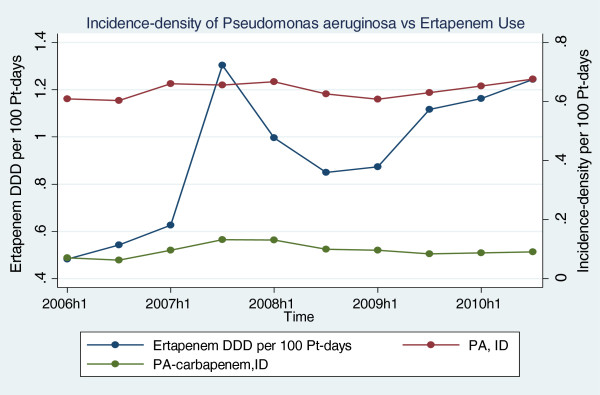
**Ertapenem Consumption against *Pseudomonas aeruginosa.***

An increase in both ertapenem-resistant *Escherichia coli* and *Klebsiella spp.* was observed between 2006 and mid-2010. Increasing ertapenem consumption correlated significantly with increasing incidence-density of ertapenem-resistant *Escherichia coli* (0.02 to 0.25 cases per 10,000 PD, R^2^ = 0.5103) and insignificantly with ertapenem-resistant *Klebsiella spp.*(0.4 to 1.0 cases per 10,000 PD, R^2^ = 0.2755) at zero time lag. The increasing trend of ertapenem consumption correlated with increasing incidence-density of carbapenem-resistant *Pseudomonas aeruginosa*, at increasing statistical trend (0.25 to 0.35 cases per 10,000 PD, R^2^ = 0.3190), at zero time lag (Figure 4).

### Correlation of ertapenem consumption with consumption of other antibiotics

**Figure 5:**
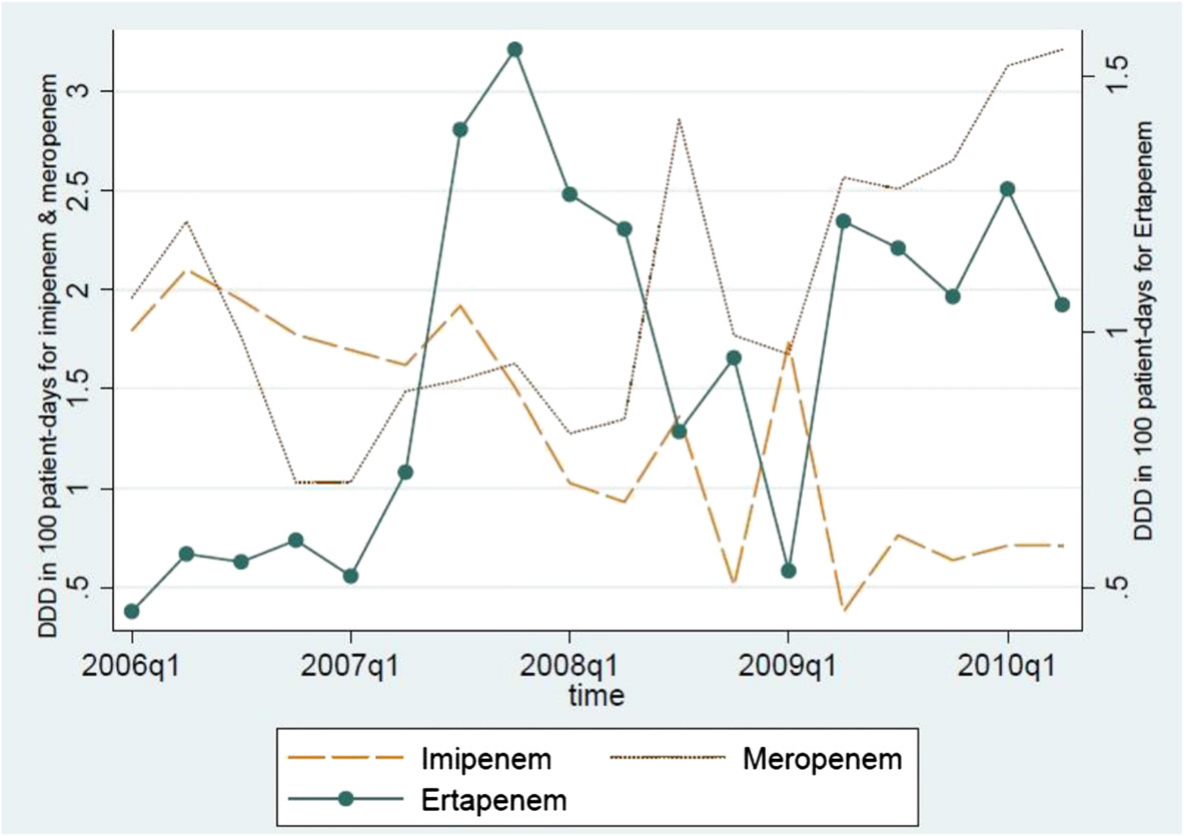
Ertapenem Consumption against Anti-Pseudomonal Carbapenem Consumption.

Ciprofloxacin use slightly increased from 1.17 DDD/100 PD in 2006 to 1.3 DDD/100 PD in mid-2010. Similarly, ceftriaxone use increased from 5.61 DDD/100 PD in 2006 to 12.5 DDD/100 PD in mid-2010. The increasing consumption of ciprofloxacin (R^2^ = 0.10093) and ceftriaxone (R^2^ = 0.22043) before, was insignificantly correlated with subsequent increasing consumption of ertapenem (Table 7). However, the increasing ertapenem consumption was correlated with subsequent decreasing consumption of cefepime 3 months later at increasing statistical trend, from 5.4 DDD/100 PD in 2006 to 4.7 DDD/100 PD (R^2^ = 0.37344). Meropenem use increased from 2.0 DDD/100 PD in 2006 to 3.2 DDD/100 PD in mid-2010, but the converse was true for imipenem, where its use decreased from 1.8 DDD/100 PD to 0.7 DDD/100 PD in mid-2010 (Figure 5). The increase in ertapenem consumption was correlated with a decrease in imipenem consumption significantly (R^2^ = 0.31081), with no time lag and was correlated with subsequent increasing consumption of meropenem (R^2^ = 0.4092) 6 months later.

**Table 7:** Correlation of ertapenem consumption with consumption of other antibiotics

**Antibiotic**	Correlation Coefficient*	**R**^2*^	P-values*	**Time Lag**
Ciprofloxacin	0.3177	0.10093	0.717	−3 months
Ceftriaxone	0.4695	0.22043	0.657	−3 months
Cefepime	−0.6111	0.37344	0.067	3 months
Imipenem	−0.5575	0.31081	0.001	Zero
Meropenem	0.2948	0.4092	0.004	6 months

*Correlation coefficient, R^2^ and P-values are obtained from cross-correlation analysis.

## Discussion

We had evaluated the appropriateness and outcomes of ertapenem use in SGH from 2006 to 2008 and assessed the impact of ertapenem on the institution’s ESBL production, and trends of gram-negative bacterial resistance, and utilization of other antibiotics from 2006 to mid-2010 (4.5 years total and 1.5 years after the evaluation of use). The rate of appropriateness of ertapenem use was high. Most of our ertapenem use was culture-directed. As cephalosporins were the most common antibiotics that were administered prior to ertapenem (47.6%), escalation from cephalosporins to ertapenem suggested that ESBL microorganisms (likely mainly ESBL producing *E. coli* in our institution) could have been selected out with its widespread use, since the increased prevalence of ESBL infections could result from an increased consumption of 3rd generation cephalosporins [22].

While ertapenem treatment success rates were high in SGH, seizures occurred in 1.0% of the patients (n = 9), which was higher than the 0.5% reported in post marketing surveillance reports [3]. This could be due to the existing concurrent medical conditions of these patients, whereby 4 out of 9 patients (44.4%) had pre-existing epilepsy history. Two patients had a history of multiple cerebral vascular accidents; another was concurrently on morphine, which may potentiate seizures [23]; and one had a recent seizure episode that may be secondary to hypoglycemia. In addition, two-third of these patients had renal impairment, which suggested that seizures may be potentiated by drug accumulation due to decreased clearance.

Primary bacteremia is an inappropriate indication for ertapenem use in our study, due to limited evidence. Secondary bacteremia from an infective source, however, is an appropriate indication. In our study, out of 172 secondary bacteremia cases, 154 cases (89.5%) had microbiological eradication. Mortality rate in these 172 cases was 13.4% (23 cases), where 6 died of other causes, and 5 others died because of concurrent infections, for example disseminated methicillin-resistant *Staphylococcus aureus* infection. Four secondary bacteremia cases treated with ertapenem were readmitted within 30 days with the same microorganism isolated; with 3 cases having poor source control previously, and 1 case was treated with only 5 days of ertapenem for ESBL *E. coli* bacteremia and subsequently oralized to ciprofloxacin despite intermediate resistance of *E. coli* to ciprofloxacin. This could have explained for the recurrence of bacteremia.

 Evidence of ertapenem efficacy in treatment of ESBL gram-negative bacteremia has been scarce so far, with only 2 retrospective studies showing favourable clinical response in treatment of both primary and secondary bacteremia [24,25]. When compared with antipseudomonal carbapenems, treatment with ertapenem for gram-negative primary bacteremia led to equivalent mortality rates and bacteriological eradication [26]. It was observed in our study that out of 57 treatment failure cases, ertapenem was used for bacteremia in 14 cases; of which, 8 were primary bacteremia cases. For patients with secondary bacteremia in our study, treatment outcomes were favourable, with a high microbiological eradication rate (89.5%) and a low mortality rate (13.4%), thereby supporting the use of ertapenem in secondary bacteremia.

There is a concern that extensive first-line use of ertapenem will select multi-drug resistant pathogens with cross-resistance to other carbapenems [5,6]. Based on our study results, increasing consumption of ertapenem correlated with subsequent increasing incidences of ertapenem-resistant *Escherichia coli* (R^2^ = 0.5103) significantly and carbapenem-resistant *Pseudomonas aeruginosa* at increasing statistical trend (R^2^ = 0.3190). This macroscopic level of observation was also validated by our observation from the detailed evaluation of 906 cases at microscopic level. While the emergence of carbapenem-resistant *Pseudomonas aeruginosa* might be due to inducible resistance after exposure to ertapenem, the emergence of ertapenem-resistant *Escherichia coli* and *Klebsiella spp.* is likely to be due to the selection of an ertapenem-resistant subpopulation (ESBLs with membrane porin loss) that already pre-exist among the ertapenem susceptible population [27]. This differed from results of 3 other studies that showed no increased incidence of carbapenem-resistant *Pseudomonas aeruginosa* isolates after introduction of ertapenem into the hospital formularies [9,10,28]. In our retrospective study, a small proportion of patients developed carbapenem-resistant *Pseudomonas aeruginosa* (2.8%), *Acinetobacter baumannii* (2.2%), and ESBL enterobacteriaceae (0.9%) within 30 days of ertapenem therapy initiation. Only data within 30 days was used for evaluation of infection recurrences in our study to prevent or reduce the impact of bacterial strains import from the community and other healthcare centres.

It was noted that the mean duration of hospital stay was 36.55 ± 45.53 days in our retrospective study. Out of 906 patients, 40 patients (4.4%) were on ertapenem for treatment of osteomyelitis or skin abscesses, with duration of ertapenem ranging from 28 to 42 days. Twenty-eight patients (3.1%) were treated for intra-abdominal abscesses, with a median duration of 24 days. The long hospital stay may have been a risk factor in the rates of carbapenem-resistant *Pseudomonas aerginosa* and *Acinetobacter baumannii* observed, as also reported by Falagas et al [29]. However, most patients were initiated on ertapenem early during hospital stay, therefore ruling out this confounding factor.

While ciprofloxacin and ceftriaxone resistance rates in *Escherichia coli* continued to increase, ciprofloxacin and ceftriaxone resistant *Klebsiella spp.* incidence densities surprisingly decreased independently, before the increase in ertapenem consumption. Hence, the increase in ertapenem consumption could be attributed to an increase in cephalosporin and ciprofloxacin resistant *Escherichia coli* mainly. An increase in incidence density of community extended-spectrum beta-lactamase (ESBL)-producing and quinolone-resistant *Escherichia coli* isolates may have contributed to this phenomenon. Pada and co-workers had found that up to 12% of more than 1,000 emergency department attendees without previous health care association at hospital 2 in 2007 were colonized with ESBL-positive enterobacteriaceae—the vast majority of which were *Escherichia coli*[30]. This was because the extensive use of ciprofloxacin in community acquired infections had probably led to a continual increase in incidences of ESBLs colonization in the community.

The increasing consumption of ertapenem correlated with subsequent increasing consumption of meropenem, which may be due to selection for Pseudomonas aeruginosa with ertapenem use. However, increasing consumption of ertapenem correlated with decreasing consumption of imipenem. This could be due to physician preference for meropenem in view of relatively lower incidence of seizures with meropenem as compared to imipenem [31-33]. The consumption of cefepime, in the treatment of ESBLs, was gradually replaced by ertapenem in our institution as there were high incidences of CTX-M producing *Klebsiella spp.* and *Escherichia coli*[34] which could have high minimum inhibitory concentrations to cefepime [35-37].

There are several limitations to this work. One of the main limitations of the study was the retrospective nature of the study. Limitations inherent to retrospective review include a dependency on previously recorded data, which may be limited by systematic or recorder bias. Since data from NARSS [15-21] is extracted primarily from the hospital microbiology and pharmacy databases, it is not possible to distinguish between colonization and infection except when there are blood culture results (i.e. “bacteremia”). In addition, the study period of 4.5 years may be too short to evaluate true impact of ertapenem on local epidemiology. Nevertheless, it still allows the determination of any broad association between ertapenem consumption and antibiotic resistance.

In this study, only SGH data on antibiotic prescription, including ertapenem, and antimicrobial resistance trends were analyzed, therefore conclusions may not be generalized to other hospitals or community. Cross-correlation analysis of ertapenem consumption and antimicrobial resistance trends was performed without adjusting for possible confounding factors, such as consumption of other antibiotics like meropenem and length of hospitalization. Infection control measures over the same period were also not assessed, which may have been a contributing factor to transmission of drug-resistant microorganisms.

## Conclusions

In conclusion, ertapenem was used appropriately in SGH, in terms of dose and indication and had resulted in a high successful treatment rate. Increasing ertapenem consumption ESBL-producing may result in the selection for multi-drug resistant bacteria, like carbapenem-resistant *Pseudomonas aeruginosa*. Hence, it would be prudent to judiciously use ertapenem for ESBL-producing microorganisms, with adequate dosage.

### Ethical approval

The Singhealth Centralized Institutional Review Board (approval number CRIB 2010/031/D) had approved this study.

## Competing interest

Kwa AL has received funding for research from Janssen-Cilag, Pfizer and Merck Sharp & Dohme (I.A.) Corp.

## Authors’ contributions

LC extracted the data, carried out the data collection and analysis for both parts of the paper and drafted the manuscript. LA, LL, NSC and WCM carried out collection and analysis of data for the first part of the paper. LW and AK designed and carried out the statistical analysis. CP and AK conceived of the study and AK helped draft the manuscript. All authors read and approved the final manuscript.

## Pre-publication history

The pre-publication history for this paper can be accessed here:

http://www.biomedcentral.com/1471-2334/13/523/prepub
